# Interleukin-33: Metabolic checkpoints, metabolic processes, and epigenetic regulation in immune cells

**DOI:** 10.3389/fimmu.2022.900826

**Published:** 2022-08-01

**Authors:** Jian Lin, Jiyun Liu, Rui Ma, Jie Hao, Yan Liang, Junjie Zhao, Ailing Zhang, Haiyang Meng, Jingli Lu

**Affiliations:** ^1^ Department of Pharmacy, The First Affiliated Hospital of Zhengzhou University, Zhengzhou, China; ^2^ Henan Engineering Research Center of Clinical Mass Spectrometry for Precision Medicine, The First Affiliated Hospital of Zhengzhou University, Zhengzhou, China; ^3^ Zhengzhou Key Laboratory of Clinical Mass Spectrometry, The First Affiliated Hospital of Zhengzhou University, Zhengzhou, China; ^4^ Fujian Provincial Key Laboratory of Innovative Drug Target Research, School of Pharmaceutical Sciences, Xiamen University, Xiamen, China

**Keywords:** IL-33, immune cells, metabolic pathways, epigenetics, metabolic checkpoints

## Abstract

Interleukin-33 (IL-33) is a pleiotropic cytokine linked to various immune cells in the innate and adaptive immune systems. Recent studies of the effects of IL-33 on immune cells are beginning to reveal its regulatory mechanisms at the levels of cellular metabolism and epigenetic modifications. In response to IL-33 stimulation, these programs are intertwined with transcriptional programs, ultimately determining the fate of immune cells. Understanding these specific molecular events will help to explain the complex role of IL-33 in immune cells, thereby guiding the development of new strategies for immune intervention. Here, we highlight recent findings that reveal how IL-33, acting as an intracellular nuclear factor or an extracellular cytokine, alters metabolic checkpoints and cellular metabolism, which coordinately contribute to cell growth and function. We also discuss recent studies supporting the role of IL-33 in epigenetic alterations and speculate about the mechanisms underlying this relationship.

## Introduction

Interleukin-33 (IL-33) is a pleiotropic cytokine that was originally described as a nuclear protein (1, 2). Intracellular IL-33 can act as a chromatin-associated nuclear factor, possessing transcriptional regulatory properties (1, 3). The precursor pro-cytokine IL-33 is processed to a mature form and released in response to cellular stress; thus, extracellular IL-33 acts as an ‘alarmin’ to alert the immune system of potential tissue stress or damage (4, 5). Extracellular IL-33 signals *via* IL-1 receptor-related protein (IL-1RL1, ST2), which shows constitutive or induced expression on most, if not all, immune cells (6, 7). Binding of IL-33 to its receptor ST2 recruits IL‐1R accessory protein (IL‐1RAP), forming the myeloid differentiation primary response protein 88 (MyD88) complex, which activates at least two independent pathways: the mitogen-activated protein kinase (MAPK) pathway and nuclear factor‐κB (NF‐κB) pathway (8, 9). A soluble form of ST2 (sST2) is released by various cell types and serves as decoy receptor to neutralize IL-33 (10). By modulating the growth and function of myeloid and lymphoid cells, IL-33 orchestrates innate inflammatory responses and shapes adaptive immunity, contributing to immune homeostasis and tissue repair in response to environmental stresses (11–13).

Of note, IL-33 can influence systemic metabolism, for example, by regulating lipid metabolism and increasing insulin production, which are correlated with changes in immunological parameters (14–16). These findings reforince the idea that cytokine-induced signals may also affect metabolic pathways; in parallel, there is clear evidence for the importance of cytokines in altering metabolic properties in immune cells, i.e., several cytokines exert anti-or pro-inflammatory effects in part *via* metabolic reprogramming (17–21). Consistent with this, IL-33-mediated metabolic programs that control the fate of immune cells are beginning to be understood. For example, the distinct IL-33-induced functional state of immune cells is accompanied by changes of peroxisome proliferator activated receptor-γ (PPAR-γ), mammalian target of rapamycin (mTOR), and hypoxia-inducible factor 1α (HIF1α) (22, 23), which are key regulators of glucose and lipid metabolism. These findings have led to interest in the role of IL-33 in a relatively new field, immunometabolism, which has flourished in the past decade (24).

Cellular metabolism not only satisfies energetic and biosynthetic requirements but can contribute to the epigenetic control of immune cell development (25, 26). As such, recent studies have suggested that IL-33 can shape the transcriptional landscape of immune cells *via* epigenetic reprogramming and chromatin accessibility (27, 28). The discovery of epigenetic modifications that mediate the effects of IL-33 provides another extensive layer of IL-33-mediated regulation in immune cells. In this review, we focus on studies of two key mechanisms by which IL-33 controls immune cells, metabolic reprogramming and epigenetic modifications, highlighting areas that we feel hold particular promise among cytokine-mediated effects. We address the possibility that alterations in metabolic checkpoints may provide a mechanistic link between cellular metabolism and immune cell development in response to IL-33. Additionally, we emphasize the role of IL-33-mediated cellular metabolism in the control of growth and effector functions of immune cells. Finally, we discuss recent discoveries and mechanisms underlying the unique epigenetic profiles of immune cells after IL-33 stimulation.

## IL-33 controls metabolic checkpoints in immune cells

**Figure 1:**
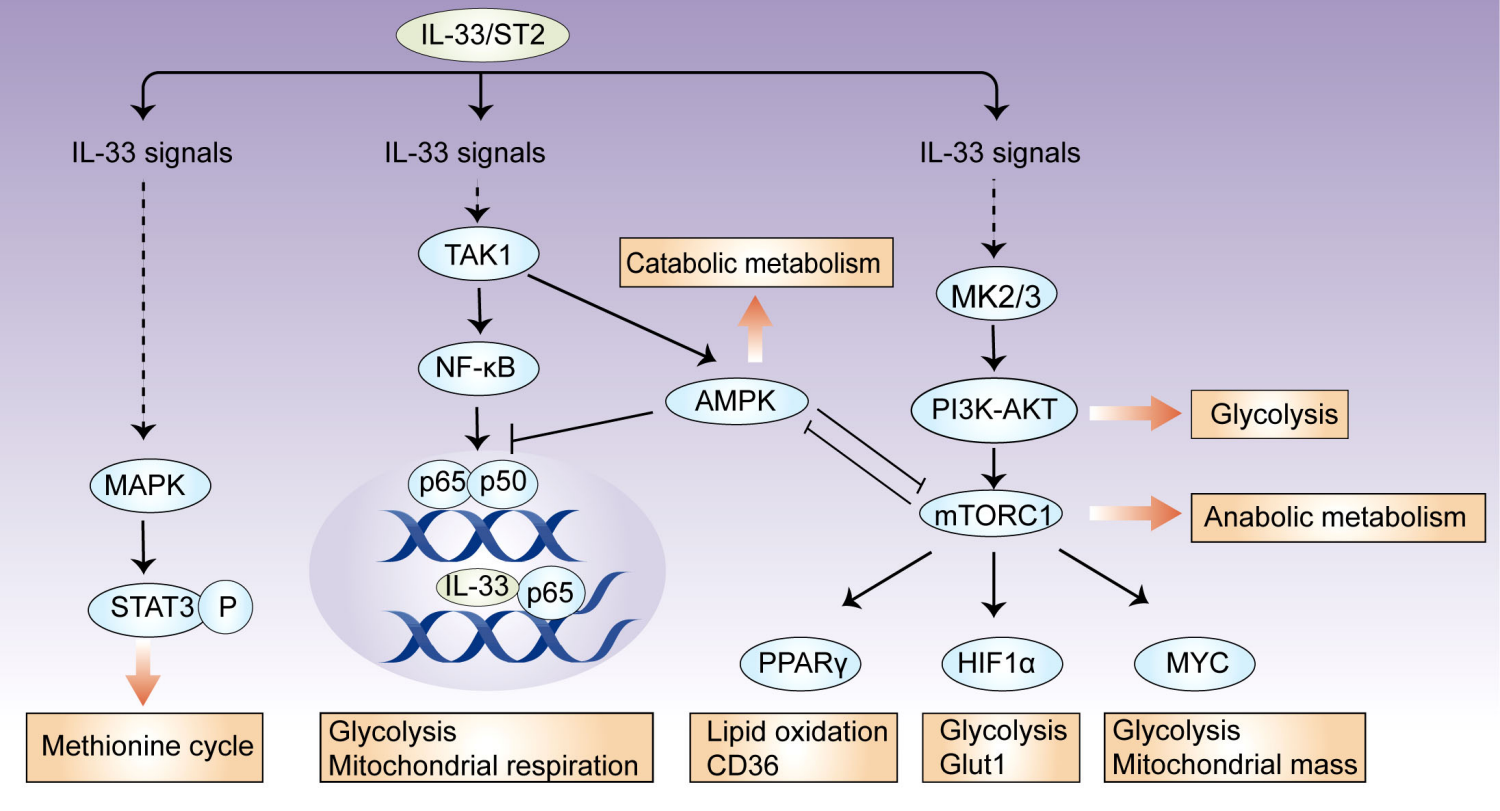
IL-33 controls metabolic checkpoints in immune cells. There are several metabolic checkpoints by which IL-33 can participate in cellular metabolism. IL-33 activates the metabolic regulators mTORC1, PI3K-AKT, and AMPK, which coordinately influence downstream effector molecules, such as HIF-1α, Myc, and PPARγ, and subsequently orchestrate glycolytic and lipogenic programs. In this process, AMPK suppresses IL-33-induced NF-κB activation; intracellular IL-33 can also bind to NF-κB p65 and restricts its transcriptional activity, suggesting that a feedback loop controls cellular metabolic homeostasis. Additionally, IL-33 activates mitochondrial STAT3 *via* MAPK and fuels the methionine cycle.

Metabolic checkpoints are involved in metabolic reprogramming for the generation of energy and metabolites, and they coordinate cellular functions by integrating environmental cues (29). In immune cells, there are several metabolic checkpoints by which IL-33 regulates cellular metabolism. Here, we focus our discussion on mTOR, AMP-activated protein kinase (AMPK), and phosphoinositide 3 kinase (PI3K)–AKT signaling (**Figure 1**).

The serine/threonine kinase mTOR comprises two complexes, mTORC1 and mTORC2, which together have key roles in cellular metabolism (30). mTORC2 mainly promotes glucose uptake and *de novo* lipogenesis, while mTORC1 orchestrates glycolytic and lipogenic programs by the induction of downstream effector molecules, such as HIF-1α, Myc, PPARγ, and SREBP1 (31, 32). In response to IL-33 stimulation, mTORC1 activity is required for increased cellular metabolism in certain immune cells. IL-33-stimulated CD8^+^ T cells require mTORC1 activity for increased rates of glycolysis, accompanied by the upregulation of *Myc* and *Hif1α* expression (22). mTORC1 activity also regulates IL-33-dependent effector functions in both Th2 cells and group 2 innate lymphoid cells (ILC2s) by a pathway that involves the upstream activator PI3K p110δ (33). In IL-33-activated ILC2s, mTORC1 controls the expression of *Pparg* and *Dgat1*, which allow the uptake of glucose and lipids, thereby fueling nutrient metabolism (23). However, IL-33-deficient Treg cells have increased phosphorylation levels of the mTORC1 complex (S6, 4E-BP1), which is closely correlated with Treg cell instability (27). Together, mTOR is an essential component of IL-33 signaling and can be activated upon immune cell exposure to extracellular IL-33; the mTOR complex can also be inhibited by intracellular IL-33, which may be associated with transcriptional repressor function of IL-33 (1, 3).

A key signaling pathway that antagonizes the mTOR-mediated control of anabolic metabolism is the metabolic sensor AMPK (34). AMPK promotes catabolic processes *via* several pathways, including the activation of PGC1α and inhibition of acetyl- CoA carboxylase 1 (ACC1) and ACC2 (35). In ILC2s, AMPK may be involved in IL-33-mediated effects and crosstalk with adiponectin (36). After interfering with AMPK expression, the ability of IL-33 to increase ILC2 cell counts is lost (37). Mechanistically, IL-33 stimulates the phosphorylation of AMPK at Thr172 in a TAK1-dependent manner (36). By inhibiting downstream pathways of TAK1, such as IKKα/β and IκBα, AMPK feedback suppresses IL-33-induced NF-κB activation and IL-13 production (36). Detailed analyses of the effects of AMPK on metabolites, mitochondrial respiration, and related metabolic pathways have not been performed in IL-33-activated immune cells, although it is well established that AMPK controls systemic thermogenesis and energy expenditure in adipose tissues *via* IL-33-activated ILC2s (36).

The PI3K–AKT signaling network influences cellular metabolism *via* many downstream effectors. They either regulate nutrient transporters and metabolic enzymes or activate downstream metabolic regulators, such as mTORC1, GSK3, and members of the FOXO family (38, 39). PI3K activity is involved in the IL-33-mediated activation of mTORC1, which, as discussed above, serves a key function in ILC2 metabolism and function (33). In response to IL-33, mast cells exhibit the MK2/3-dependent activation of ERK1/2, which consequently stimulates PI3K–AKT signaling, leading to cytokine production and leukocyte attraction (40).

As discussed, several metabolic regulators involved in the activation and function of IL-33-stimulated immune cells have been identified. Furthermore, studies have begun to reveal their contribution to the IL-33-mediated reprogramming of metabolic processes, although more studies are needed. The specific mechanisms by which these metabolic checkpoints coordinately and differentially regulate metabolic processes during in different immune cell states in response to IL-33 are unknown.

## Regulation of cellular metabolism by IL-33

The metabolic checkpoints discussed above work together to modulate cellular metabolism, which determines cell growth, survival, and function. Although it is not clear how IL-33 is coupled to metabolic checkpoints, multiple studies have begun to demonstrate how IL-33 regulates cellular metabolic processes to meet innate metabolic demands for cell activation and functions.

### Regulation of ILC2 metabolism by IL-33

ILCs are a novel lymphocyte subfamily; they express the characteristic surface receptors and effector molecules of differentiated T cell subsets under the control of specific transcription factors (41). Among them, ILC2s express GATA3 and ST2; as such, they react rapidly to IL-33 and produce type 2 cytokines, including IL-5 and IL-13 (42). ILC2 activation by IL‐33 not only enables them to support type 2 immune responses but to facilitate tissue repair by the production of amphiregulin and the generation of reparative M2-like macrophages (15, 43).

Following the activation of IL-33, ILC2s become highly proliferative and require an elevated glycolytic capacity to produce IL-13 (44, 45). However, a shift from oxidative phosphorylation (OXPHOS) toward increased glycolysis leads to defective ILC2 maturation and function (46, 47). IL-33-stimulated ILC2s also require glutamine to fuel OXPHOS and maintain cell function and proliferation (45). This could be partially explained by the observation that glycolysis results in attenuated ST2 expression (46), indicating negative feedback between IL-33/ST2 signaling and cell-intrinsic glycolytic metabolism in ILC2s. In addition to glycolysis, IL-33 also enhances the mitochondrial membrane potential and ATP synthesis in ILC2s and promotes ILC2-driven allergic inflammation in the lung (48). Consistent with this, IL-33 activation has been shown to induce reactive oxygen species (ROS) production by mitochondria, and ROS are required for optimal IL-33-triggered activation of metabolic processes in ILC2s (49). ROS scavengers can reduce the production of IL-5 and IL-13, ILC2 proliferation, and ILC2-mediated eosinophilia in response to IL-33 stimulation (49).

These metabolic programs coordinate the uptake of environmental nutrients, as IL-33 increases glucose and fatty acid uptake to promote the formation of lipid droplets in ILC2s (23). In this context, the availability of glucose allows the uptake and storage of external lipids, and both functions are required to fuel the proliferation of ILC2s (23). This cross-regulation of glucose and fatty acid metabolism may be mediated by mTOR, which controls the expression of *Pparg* and *Dgat1* (23). Of note, DGAT1, an enzyme involved in lipid droplet formation, increases the uptake of external lipids and protects ILC2s from lipotoxicity (23). Thus, it is reasonable to conclude that IL-33-mediated processes that give rise to functional ILC2 are dependent on metabolic reprogramming, which integrates metabolic pathways with nutrient availability.

**Figure 2:**
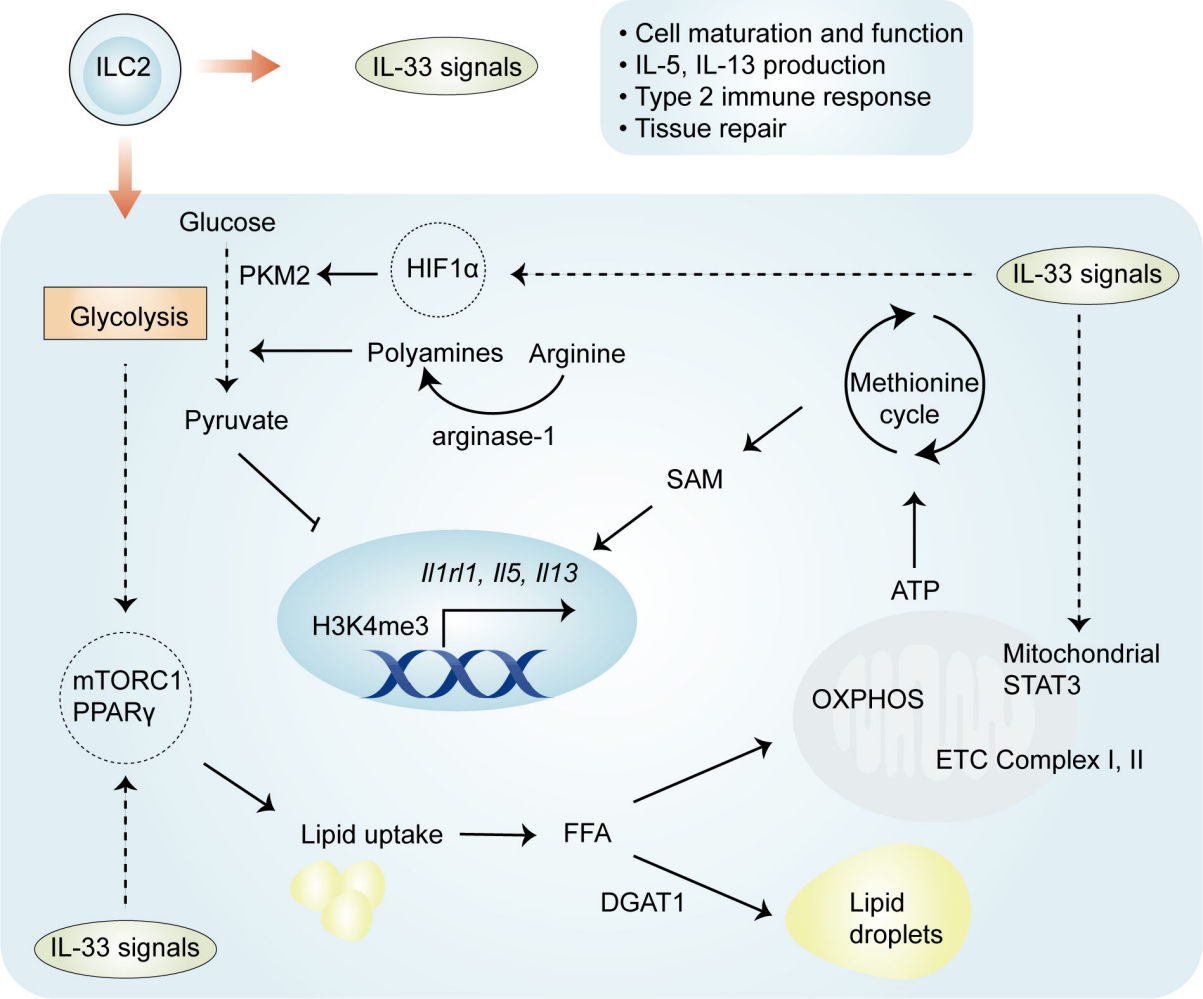
Regulation of ILC2 metabolism by IL-33. Both glycolysis and OXPHOS are activated in IL-33-activated ILC2s, and the glycolytic process allows the uptake and storage of external lipids to fuel the proliferation of ILC2s. Four important regulators—HIF-1α, STAT3, arginase-1, and PPARγ—have been implicated in IL-33-activated ILC2s. HIF-1α drives the expression of the glycolytic enzyme PKM2 and the glycolytic metabolite pyruvate, which reduce H3K4me3 levels at ILC2-specific genes. IL-33 drives the activation of STAT3 and subsequent generation of SAM, which leads to increased H3K4me3 levels. IL-33 also increases the expression of PPARγ, which mediates lipid metabolism in ILC2s. The enzyme arginase-1 promotes the generation of l-arginine-derived polyamines and is closely related to aerobic glycolysis in IL-33-activated ILC2s. OXPHOS, oxidative phosphorylation; PKM2, pyruvate kinase M2; SAM, S-adenosylmethionine.

Mechanistically, four important regulators—HIF-1α, STAT3, arginase-1, and PPARγ—have distinct but crucial roles in the regulation of metabolic pathways involved in IL-33-mediated ILC2 proliferation and function (**Figure 2**). Recently, it has been elegantly shown that IL-33-activated mitochondrial STAT3 is required for ATP production to fuel the methionine cycle and generate S-adenosylmethionine (SAM), shaping ILC2 effector function (48). These results are at least partially consistent with those obtained in other cells (50, 51); mitochondrial STAT3, a modulator of mitochondrial respiration, can sustain OXPHOS and ATP production by regulating the activities of complexes I and II of the electron transport chain (50, 51). The production of SAM by STAT3 activation leads to increased levels of H3K4me3, a transcriptional permissive modification, at the *Il5* and *Il13* loci (48). These results suggest that the role of IL-33 in ILC2s is complex and involves an immuno-metabolite-epigenetic axis. This is further supported by the finding that in IL-33-activated ILC2s, HIF-1α accumulation results in enhanced glycolytic capacity and attenuated mitochondrial respiration (46). In particular, HIF-1α drives the expression of the glycolytic enzyme pyruvate kinase M2 (PKM2) and glycolytic metabolite pyruvate, with a central role in controlling the homeostasis of ILC2s (46). PKM2-pyruvate metabolic checkpoint reduces H3K4me3 at the *St2* and *Il5* loci as well as at the *Gata3* promoter in ILC2s with increased glycolytic capacity (46). As discussed below, these findings indicate that IL-33-mediated cellular metabolism promotes energy production, and metabolic intermediates can act as epigenetic regulators.

The enzyme arginase-1 metabolizes the amino acid l-arginine to generate urea and ornithine (52) and is selectively expressed by ST2^+^ ILC2 populations (44). This enzyme is also upregulated in IL-33-expresing myeloid-derived suppressor cells (MDSCs) (28). Arginase-1 enzymatic activity serves as an essential checkpoint controlling IL-33-activated ILC2 metabolism, subsequently contributing to cell proliferation and the development of type 2 inflammation (44). Through its role in metabolizing arginine, the disruption of arginase-1 in ILC2s prevents the generation of l-arginine-derived polyamines (44), molecules that support cell growth and survival (53). In addition to the above effects, other studies have pointed to the role of l-arginine in glycolytic function, without altering mitochondrial biogenesis or the function of activated T cells (54). Consistent with the effects of l-arginine in T cells, arginase-1 is closely related to aerobic glycolysis in IL-33-stimulated ILC2s (44). The inhibition of arginase-1 enzymatic activity does not affect OXPHOS but markedly reduces the maximal glycolytic capacity, thereby affecting proliferation (44). These studies demonstrate an essential role for arginase-1 enzymatic activity in IL-33-induced ILC2 proliferation.

The metabolic sensor PPARγ is selectively expressed in ILC2s, and a PPARγ deficiency intrinsically impairs ILC2 function (55, 56). The activation of ILC2 by IL-33 leads to the increased expression of PPARγ and, in turn, PPARγ upregulates ST2 expression on ILC2s (55, 56), suggesting that there is a positive feedback loop between PPARγ and IL-33/ST2 signaling in the regulation of ILC2 activation. PPARγ has a crucial role in various aspects of IL-33-mediated ILC2 proliferation and function. First, the pharmacologic inhibition or genetic deletion of PPARγ in ILC2s significantly impairs IL-33-induced mitochondrial fitness, which subsequently results in reductions in IL-13 and IL-5 secretion (57). Second, the pharmacological inhibition of PPARγ leads to decreased expression of CD36, which transports particular lipids into ILC2s for conversion into PPARγ ligands and facilitates fatty acid uptake (55). Third, the inhibition or absence of PPARγ also reduces glucose uptake (55), a likely consequence of the modulation by PPARγ-mediated glucose transporter expression (58). Of note, the expression of *Pparg* can be controlled by the availability of glucose, particularly *via* the nutrient sensor mTOR in ILC2s (23). Thus, IL-33-activated ILC2s depend on PPARγ, which controls glucose and fatty acid metabolism.

Collectively, these studies indicate that IL-33/ST2 signaling can regulate various metabolic regulators that adapt to intrinsic metabolic demands of ILC2s. By the regulation of metabolic pathways, IL-33-treated ILC2s promote airway hyperreactivity and lung inflammation (47, 49, 59) and drive pro-tumorigenic immunity (57). These studies provide a foundation for further investigations of strategies to modify ILC2 metabolism for disease treatment.

### Regulation of Th2 cell metabolism by IL-33

Like ILC2s, Th2 cells constitutively express high levels of ST2 and respond directly to IL-33, thereby producing Th2-related cytokines IL-5 and IL-13 and effectively inducing type 2 immunity (60). With activation *via* IL-33, Th2 cells exhibit a high spare respiratory capacity and high extracellular acidification rate, although both rates are lower than those observed in ILC2s (44). It is not clear whether these findings reflect fundamental differences between the metabolic pathways in ILC2s and Th2 cells or differences in the intensity or duration of type 2 immunity. Nevertheless, Th2 cells share the same PPARγ expression patterns as those in ILC2s (61, 62), and this expression is critical for ST2 expression in Th2 cells (62). In these contexts, IL-33-activated Th2 cells promote type 2 inflammatory responses in allergic airway inflammation and anti-infection responses (61, 62). Furthermore, consistent with IL-33 in ILC2s, IL-33-induced IL-5 and IL-13 production by Th2 cells also depends on mTOR activation (33). Altogether, Th2 cells and ILC2s, as important effector cells and sources of type 2 cytokines during the inflammatory process, have shared metabolic pathways and metabolic profiles in response to IL-33 stimulation. However, our understanding of the roles of IL-33 in Th2 cell metabolism during type 2 immunity remains at an early stage, and further studies are needed.

### Regulation of macrophage metabolism by IL-33

Macrophages are important components of the innate system and have unique tissue-specific functions (63). Depending on the developmental origin, location, and microenvironmental cues, macrophages exhibit extensive phenotypic and functional plasticity with a wide range of roles in homeostatic and pathological conditions (64). Despite the high diversity in these populations, it is important to note that some data related to IL-33 are contextualized within the classical M1-M2 macrophage polarization system (64–67). Studies have revealed that IL-33 can contribute to macrophage polarization and metabolic processes in both pro-M1 and pro-M2 settings (65–67). In the pro-M1 setting stimulated with LPS, IL-33 overexpression promotes glycolysis and decreases mitochondrial function (68), consistent with the fact that M1 macrophages preferentially utilize aerobic glycolysis (69). Accordingly, an IL-33/ST2 signaling deficiency increases the number and activity of mitochondria in macrophages; this can be attributed to high expression levels of *Ppargc1a*, which encodes PGC-1a, a master regulator of mitochondrial biogenesis (68). In this context, *St2*-deficient macrophages have decreased *Il1a*, *Il1b*, *Nos2*, and *Ifng* expression as well as IL-1α, IL-1β, and IFNγ production (68). M2 macrophages, which are induced by stimulation with IL-4 and IL-13, exhibited the marked upregulation of fatty acid oxidation (FAO) and OXPHOS (70). A similar metabolic process occurs in IL-33-activated macrophages under the pro-M2 setting. IL-33 overexpression decreases glycolysis but increases OXPHOS, accompanied by increased M2 marker gene expression (71). This metabolic shift is due to increased mitochondria and consequently decreased mitochondrial autophagy, which is associated with IL-33-mediated mTOR activity (71). These opposing results led to the hypothesis that IL-33 contributes to metabolic divergence among macrophage lineages in cooperation with other signaling factors. Of note, since these results stem from studies of extremely polarized macrophages generated *in vitro* under a defined inflammatory condition, the simplification of the M1/M2 paradigm makes it difficult to clearly determine how IL-33 drives the metabolic process in the specific microenvironmental niche.

**Figure 3:**
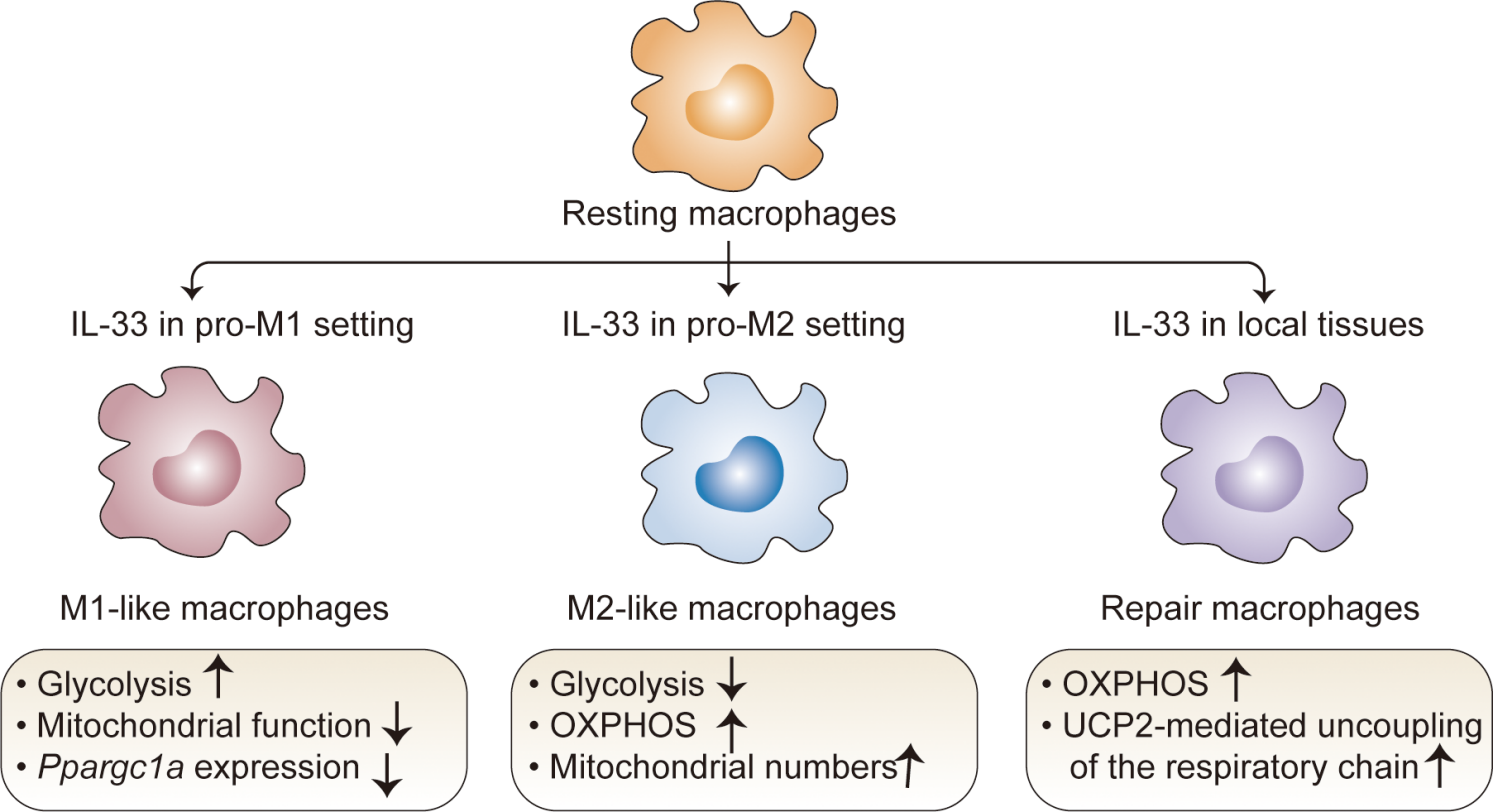
Regulation of macrophage metabolism by IL-33. In the pro-M1 setting, IL-33 drives glycolysis and decreases mitochondrial function; in the pro-M2 setting, IL-33 decreases glycolysis but increases OXPHOS. IL-33 alone directs macrophages toward a M2-like state, which mainly use OXPHOS to increase basal respiration and ATP production; in this context, UCP2-mediated uncoupling of the respiratory chain plays a critical role, which blocks the generation of ROS and allows sustained mitochondrial respiration and an intact TCA cycle. OXPHOS, oxidative phosphorylation; TCA, tricarboxylic acid; UCP2, uncoupling protein 2.

Recent studies with different animal models have revealed the metabolic control of IL-33/ST2 signaling in tissue-resident macrophages with tissue-specific transcriptional signatures and functions (72, 73). IL-33 alone can directly poise macrophages for differentiation toward a ‘tissue-reparative’ and M2-like state, which promotes muscle regeneration (72) and protects against chronic rejection in cardiac transplants (73). In these contexts, IL-33 controls rapid metabolic rewiring during macrophage development (73). At early time points following IL-33 stimulation (6 h), IL-33 does not directly affect the extracellular acidification rate and does not change the basal or maximal respiratory capacity in macrophages (72). At later time points following IL-33 stimulation (15–18 h), IL-33-activated macrophages mainly use OXPHOS to increase basal respiration and ATP production; they metabolize fatty acids and limit anaerobic glycolysis (73). This phenomenon is consistent with increased concentrations of carnitine (73), which is required for fatty acid transport into the mitochondria (73, 74), as well as increased concentrations of aspartate, malate, and fumarate (73), which are components of the aspartate-argininosuccinate shunt (AASS) and coordinate with the tricarboxylic acid cycle (TCA) cycle (75–77). These results provide insight into metabolic kinetics in macrophages following IL-33 induction. This could also be explained by the observation that IL-33 sequentially triggers a molecular transition from a pro-inflammatory to a pro-resolving M2-like macrophage phenotype in response to tissue damage-related signals (72). Mechanistically, uncoupling protein 2 (UCP2)-mediated uncoupling of the respiratory chain has a critical role in the response to the IL-33-induced metabolic profile in macrophages, which blocks the generation of ROS and allows sustained mitochondrial respiration and an intact TCA cycle (72). Of particular interest is the IL-33-dependent increase in the mitochondria-derived metabolite itaconate, which activates the transcription factor Nrf2, subsequently triggering GATA3-mediated M2-like macrophage polarization (72). These results establish the important role of an immune-metabolic axis during the transition from pro-inflammatory monocytes to anti-inflammatory macrophages upon tissue injury (**Figure 3**).

These findings provide a brief view of the diverse metabolic processes of macrophages that are regulated by IL-33 under different environmental cues, conferring functional specialization. An understanding of the metabolic state of IL-33-activated macrophages is required to fully understand the complexity of the interplay with the specific immune microenvironment and with the evolving process of cellular differentiation.

### Regulation of Treg cell metabolism by IL-33

Regulatory T cells (Treg cells) expressing the Foxp3 transcription factor ensure immune homeostasis by the control of tissue- and inflammation-specific responses (78). In the context of autoimmunity and inflammation, extracellular IL-33 binds to ST2 directly to promote local expansion, stability, and the conversion of CD4^+^Foxp3^-^ T cells to Foxp3-expressing inducible Treg cells in non-lymphoid tissues (adipose tissue, skeletal muscle, and colon) (79–81). Interestingly, this phenomenon is not observed in Treg cells within the tumor microenvironment (27); intratumoral Treg cells need intranuclear IL-33 to shape their transcriptional landscape and maintain their suppressive properties (27). In this regard, IL-33 has the ability to shape the plasticity and heterogeneity of Treg cell compartments by distinct anatomic locations and inflammatory environments.

Consequently, IL-33-activated Treg cells have unique metabolic profiles that confer location-specific properties by activating metabolic transcriptional factors and pathways. In muscle Treg cells, extracellular IL-33 induces the expression of genes typically associated with metabolism, such as *Pfkfb1* (which encodes 6-phosphofructo-2-kinase/fructose-2,6-biphosphatase 1), *Adh1* (which encodes alcohol dehydrogenase), *Fbp2* (which encodes fructose-bisphosphatase 2), and *Vldlr* (which encodes very low-density lipoprotein receptor) (80), and this may reflect metabolic responses induced by the IL-33-ST2 axis. In this study, IL-33-expressing Treg cells accumulate in injured muscles of old mice and promote muscle regeneration (80). In adipose tissue, Treg cells are tightly associated with improvements in metabolic parameters in obese mice and uniquely express PPARγ, which induces lipid metabolism (79). This is consistent with the fact that Treg cells favor FAO-driven OXPHOS, which maintains the suppressive phenotype and is further promoted by the expression of Foxp3 (82–84). However, in this context, IL-33 acts as an accessory to Treg cell metabolism, rather a driving force; TCR signaling (and not IL-33) directly upregulates *Pparg* (79). At this point, the molecular mechanism underlying the effects of extracellular IL-33 on Treg cell metabolism are unknown; however, it is interesting to speculate that PI3K-Akt-mTORC1 signaling may be involved. IL-33-mediated Treg cell differentiation requires the adaptor protein MyD88, which is a putative downstream adaptor protein in Toll-like receptor (TLR) signaling (85). TLR signaling increases glycolysis and the expression of Glut1 in Treg cells *via* PI3K-Akt-mTORC1 signaling and impairs the Treg cell suppressive capacity (83). These findings are at odds with the fact that mTOR can coordinate transcriptional programs and mitochondrial metabolism in activated Treg cells to promote immune tolerance and tissue homeostasis (86). This discrepancy may point to the intriguing possibility that the effects of IL-33 on Treg cell functions may be determined by the extent to which IL-33 interacts with these metabolic pathways.

In intratumoral Treg cells under a nutrient deficiency and sufficient immunosuppressive metabolites, intranuclear IL-33 can employ a cell-intrinsic role to shape the function and metabolic profile of Treg cells to promote tumor development (27). IL-33-deficient Treg cells exhibit an increased abundance of phosphorylated mTOR, S6, and eukaryotic translation initiation factor 4E-binding protein 1 (4E-BP1) (27). mTOR appears to antagonize Treg cell differentiation and expansion *in vitro* and suppressive activity *in vivo* (87), consistent with a ‘fragile’ phenotype of intratumoral Treg cells in the absence of IL-33 (27). Moreover, the repression of NF-κB by intranuclear IL-33 may also contribute to the metabolic profile of intratumoral Treg cells (27), as NF-κB inhibition induces the utilization of glycolysis and impairs mitochondrial respiration (88). IL-33 is therefore related to the diversity of metabolic signaling that can be exploited for the phenotypic and functional specialization of Treg cells; however, more studies are needed to fully characterize metabolism in IL-33-expressing Treg cells. For example, it is not clear how these signaling pathways are intertwined with extracellular nutrients and metabolites and how they shape cell-intrinsic metabolic programming and ultimately determine the fate of IL-33-expressing Treg cells.

### Regulation of CD8^+^T cell metabolism by IL-33

Cytotoxic CD8^+^T cells help to eliminate intracellular infections and kill malignant cells, while memory CD8^+^ T cells provide long-term protective immunity from reinfection (89). The differentiation of naïve CD8^+^ T cells into effector and memory T cell populations involves profound and unique metabolic reprogramming (89). As virus-specific CD8^+^T cells express the IL-33 receptor, IL-33 enhances the conventional memory CD8^+^T cell response and promotes their expansion following virus infection *via* the IL-33-ST2 axis (90, 91).

In IL-33-activated CD8^+^T cells, by the control of mTORC1 activity, IL-33 increases glucose uptake and lactate production, leading to vigorous effector responses to LCMV infection (22). This metabolic process is associated with the upregulation of *Glut1* expression, glycolytic enzymes, and key regulators, including *Myc* and *Hif1α* (22). In these cells, IL-33 fails to directly alter mitochondrial functions, as evidenced by the lack of an increase in mitochondrial membrane potential by IL-33 treatment (22). Given the highly proliferative nature of IL-33-expressing CD8^+^ T cells, it may not be surprising that they promote glycolysis in this context, which highlights the rapid production of energy that is necessary to fuel proliferation and differentiation.

It seems paradoxical that a recent report by Cupovic et al. revealed a dominant role of IL-33 in modulating mitochondrial morphology and the maintenance of metabolic fitness in inflating memory CD8^+^T cells (92); this study revealed that adenovirus-based vaccination confers a protective effect and contributes to the metabolic fitness of inflating memory CD8^+^T cells in an IL-33-dependent manner (92). Mechanistically, inflating memory CD8^+^ T cells deficient in IL-33 lose the typical inflating-memory phenotype and function and show marked losses in mitochondrial maintenance and activity, with reductions in both mitochondrial membrane potential and the expression of electron transport chain components, including *Uqcrc2* (Cytochrome b-c1 complex subunit 2), *Sdha* (Succinate dehydrogenase complex subunit A), and *Cox 4I1* (Cytochrome c oxidase subunit 4 isoform 1) (92). In agreement with the energy requirement of memory CD8^+^T cell differentiation, mitochondrial function impacts ETC complex formation and thus the ability of memory CD8^+^ T cells to use long-chain fatty acid β-oxidation to sustain a high spare respiratory capacity (93, 94). As discussed above, IL-33 has been implicated in glycolysis in the activation of CD8^+^T cells and, of note, is also important in maintaining mitochondrial dynamics during the differentiation of memory CD8^+^T cells following virus infection.

### Regulation of NK cell metabolism by IL-33

Natural killer (NK) cells are important innate lymphocytes with rapid cytolytic activity in infectious diseases and cancer (95). Resting NK cells have relatively low basal metabolic rates, while activated NK cells have elevated glycolysis and OXPHOS rates, and these processes are closely coupled with their effector functions (96). Although IL-33 has the ability to promote NK cell proliferation and activation (97), the metabolic functions of IL-33 in NK cells are not well defined. IL-33 is not considered a major metabolic regulator in NK cells. IL-33 can induce the expression of transferrin receptor CD71 and the l-amino acid transporter CD98 (SLC3A2) in NK cells *via* MyD88 signaling (98). This effect is partly partially reliant on IL-12 (98), which can increase the expression of ST2, rendering NK cells sensitive to IL-33 activity (99–101). It is also worth noting that IL-33 has an indirectly deleterious effect on NK cell function by metabolically modulating the tumor microenvironment (102). The effect of IL-33 is dependent on ILC2-driven lung eosinophilia, which restricts extracellular glucose availability and impairs the glycolysis-dependent effector functions of lung NK cells (102). However, extensive work is needed because the potential for direct effects of IL-33 on the NK cell metabolic profile has not been determined.

### Regulation of mast cell metabolism by IL-33

Mast cells can function as effector and immunoregulatory cells in IgE-associated immune responses (103). IL-33 activates ERK phosphorylation and NF-kB-mediated transcription, leading to an increase in the rates of glycolysis and OXPHOS in mast cells (104). Interestingly, it is glycolysis and not OXPHOS that directly enhances mast cell production of IL-6 and TNF by modulating ATP production (104). Glycolytic inhibitors suppress IL-33-induced mast cell function, evidenced by decreased neutrophil recruitment and cytokine production in an animal model of peritonitis, which highlights the more critical role of the rapid production of energy in initial mast cell activation over OXPHOS (104). Of note, this effect can also be inhibited by activating AMPK, an important metabolic regulator that induces a switch from anabolic to catabolic pathways, indicating an antagonistic metabolic effect between IL-33 and AMPK (104). Increased lactic acid is also a key feature of IL-33-activated mast cells (104). By contrast, lactic acid can selectively alter IL-33 signaling, including suppressed TAK1, JNK, ERK, and NF-κB phosphorylation, accompanied by increased HIF-1α expression, contributing to the suppression of IL-33-induced inflammatory cytokine and chemokine secretion (105). Collectively, these data suggest that a feedback loop involving the IL-33-glycolysis-lactic acid axis regulates the activation of mast cells.

### Regulation of eosinophil metabolism by IL-33

Eosinophils have multiple functions in the innate immune system, with display key effector functions in allergic diseases, helminth infections, and cancers (106, 107). IL-33 signaling *via* ST2 is not only crucial for eosinophil activation and accumulation/migration but also promotes the formation of an active degranulating synapse for eosinophil effector functions (107). With respect to metabolism, eosinophils are associated with IL-33-driven energy expenditure and browning of white adipose tissue in obesity (108). A lipidomic analysis of inflamed lung tissues has further identified a role of 12/15-lipoxygenase-derived lipid mediators in IL-33-induced eosinophilic airway inflammation, as demonstrated by the prevention of inflammation after administration of 14(*S*)-HDoHE, a major product of 12/15-lipoxygenase (109). Importantly, a transcriptome analysis of eosinophils has revealed that the IL-33-dependent secretion of IL-4 and IL-13 is likely associated with the increased expression hypoxia- and glycolysis-related genes, which are increased by the surface expression of sialic acid-binding immunoglobulin-like lectin F (Siglec-F), a lineage-specific marker of eosinophils in mice (110). These upregulated genes include hypoxia-associated genes (*Fam162a*, *Egln2*, *Ankrd37*, *Hilpda*, *Ftl1*, and *Prdx1*), likely hypoxia-induced metabolism/glycolysis-associated genes (*Pfkl*, *Tpi1*, *Pgk1*, and *Pgm2*), and genes encoding the hypoxia-induced, proinflammatory cytokine migration inhibitory factor (110). However, the metabolic features (e.g., metabolic profiles and key metabolic parameters, such as glycolysis and OXPHOS) of eosinophils mediated by IL-33 have not been comprehensively examined.

## Epigenetic modifications in immune cells by IL-33

Herein, we use the term ‘epigenetic modification’ to refer to mechanisms that can alter gene expression in the context of the same DNA sequence (111). Epigenetic regulation provides an explanation for molecular rewiring in cytokine-polarized cells, justifying the need to better understand epigenetic changes in immune cells in response to IL-33. The mechanisms by which IL-33 is coupled to epigenetic programming in immune cells are not well understand.

**Figure 4:**
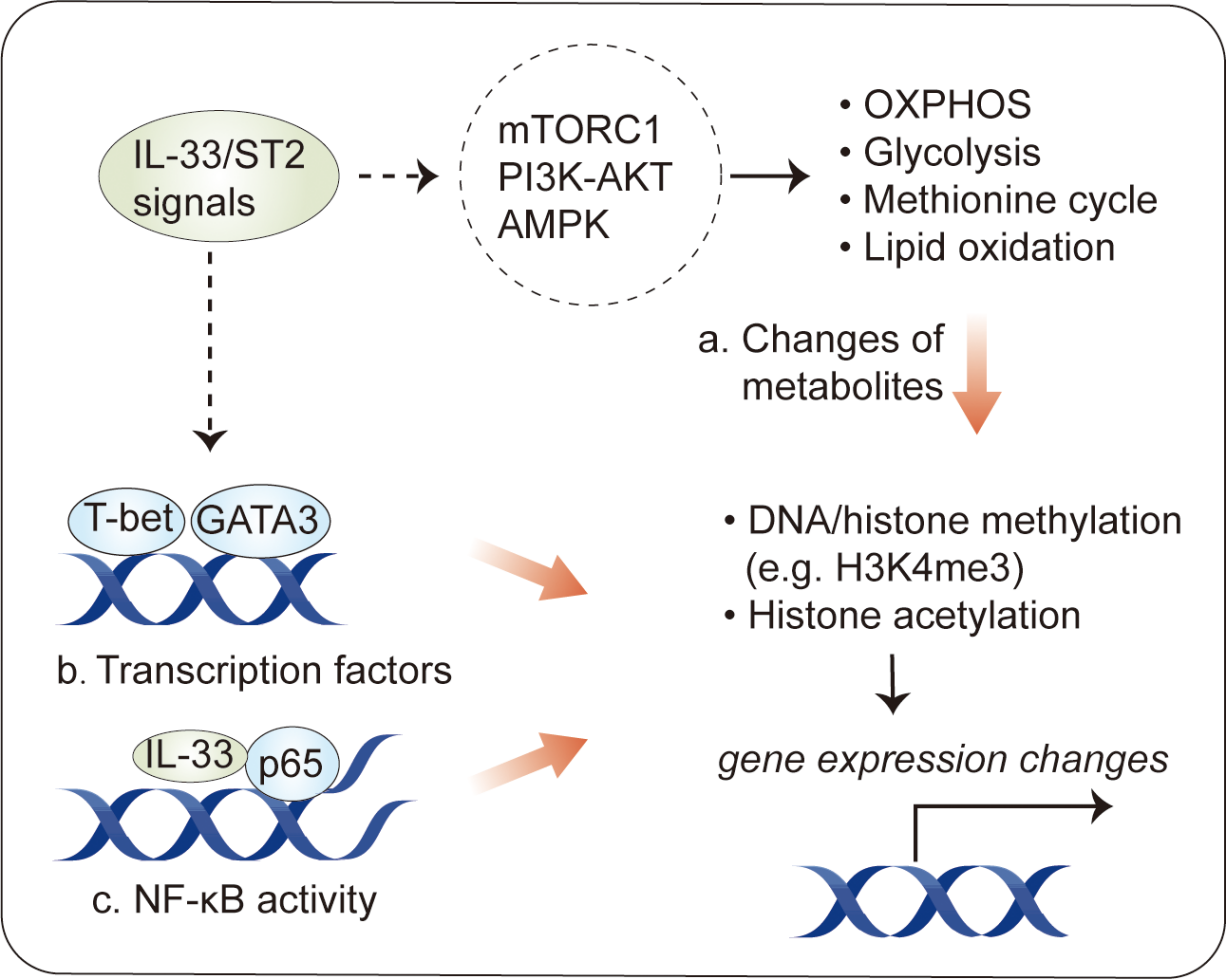
Epigenetic modification in immune cells by IL-33. Epigenetic changes have been described in IL-33-activated immune cells. **(A)** One consequence of the metabolic changes induced by IL-33 stimulation is an alteration in the production of intermediate metabolites, some of which influence transcriptional activity by the epigenetic regulation of DNA or histones. **(B)** IL-33 activates transcription factors, such as GATA3 and T-bet, which can induce epigenetic changes at target loci. **(C)** Intracellular IL-33, as a chromatin-associated nuclear factor, can bind to NF-κB p65 to dampen NF-κB-stimulated gene transcription.

Recently, several studies have revealed epigenetic modifications following by IL-33 stimulation in immune cells. In MDSCs, IL-33 induces histone H3 Lys-4 trimethylation (H3K4me3) and histone H3 Lys-14 acetylation (H3K14ac) but decreases the levels of H3K18ac (28). IL-33 does not have a marked effect on H3K9ac or H3K27me3, a stable histone mark associated with gene repression (28). In Th2 cells, IL-33 increases H3K4 trimethylation and H3K9 acetylation and decreases H3K27 trimethylation at the *Il5* locus, enhancing IL-5 production (112). Tissue-resident Treg cells expressing ST2 have distinct methylome profiles, with 11,000 differentially methylated regions associated with about 4,000 genes (113), suggesting that there is an association between epigenetic profiles and characteristics in IL-33-related immune cells. However, the mechanisms dictating epigenetic modifications induced by IL-33 are far from complete (**Figure 4**).

One consequence of the metabolic changes in response to IL-33 stimulation is an alteration in the production of metabolites (44, 48). Some metabolites can be used as substrates for epigenetic modifications, such as acetyl-CoA for histone acetylation and α-ketoglutarate for demethylases (111). The existence of metabolite–epigenetic links may help to explain the role of IL-33 in epigenetic modifications. This hypothesis is supported by results obtained for IL-33-stimulated ILC2s. As mentioned above, IL-33 increases the glycolytic capacity in ILC2s (44). *Via* the PKM2-pyruvate checkpoint, glycolysis leads to a decrease in H3K4me3 modification, a transcriptional permissive modification, at *Il1rl1* (*ST2*) (46), resulting in a negative feedback loop controlling IL-33-mediated ILC2 maturation. In addition to glycolysis, IL-33-mediated STAT3 activity, which increases the levels of SAM, a major methyl donor during DNA or histone methylation, can dramatically increase H3K4me3 levels at *Il5* and *Il13* (48). This is a canonical example of IL-33-regulated metabolites acting in concert to regulate epigenetic marks in immune cells.

Furthermore, several studies have identified transcription factors, such as GATA3 and T-bet, as a possible initiator of epigenetic remodeling in immune cells (114–116), suggesting that these transcription factors may cooperate in IL-33-stimulated epigenetic modification. Supporting this hypothesis, IL-33-induced GATA3 phosphorylation has been described in Treg cells and Th2 cells (117); GATA3 activity leads to histone H3K4 methylation and H3K9 acetylation at Th2 cytokine genes, such as *Il4*, *Il5*, and *Il13* (115, 118). Of interest, it has recently been shown that IL-33 induces ERK1/2 kinases to increase chromatin accessibility for GATA3 motifs during the development of red pulp macrophages (119). In addition to GATA3, IL-33 promotes the Th1 lineage-specifying transcription factor T-bet (120, 121), which induces epigenetic changes through physical interactions with epigenetic modifying complexes, particularly the H3K27-demethylase JMJD3 and the H3K4-methyltransferase Set7/9 complex, and target genes (116, 122, 123).

Finally, intracellular IL-33, acting as a chromatin-associated nuclear factor, possesses transcriptional repressor properties (1). Consistent with this, intracellular IL-33 can bind to NF-kB p65 to dampen NF-κB-stimulated gene transcription (3); however, it is not clear whether this explains the ability of intracellular IL-33 to modulate epigenetic profiles in immune cells. It is interesting to note that IL-33-deficient Treg cells, which attenuate suppressive properties in an ST2-independent fashion, exhibit epigenetic reprogramming with increased chromatin accessibility at the *Ifng* locus in an NF-κB–T-bet-dependent manner (27).

As discussed above, both extracellular and intracellular IL-33 participate in epigenetic modifications in an ST2-depednent or independent manner and are important in immune cell responses to IL-33, although formal proof of the underlying mechanism is lacking.

## Perspectives

Over the past decade, our understanding of cellular responses to IL-33 has expanded substantially beyond its induction of the core MyD88/MAPK/NF‐κB signaling pathways as well as its intrinsic role (i.e., chromatin-associated transcriptional repressor properties). We are now realizing that IL-33 can induce a complex cell state by epigenetic and metabolic mechanisms in immune cells. This is of note, given the recent interest in the link between epigenetic reprogramming and immune cell metabolism. However, as a relatively new area of IL-33 biology, many unresolved issues remain.

It is now clear that each immune cell subset has distinct metabolic properties that are in accord with functional demands in the context of specific challenges. Briefly, aerobic glycolysis drives rapid ATP production for cell proliferation and activation, whereas OXPHOS is required for energy-intensive processes in regulatory and memory properties (124). These metabolic pathways are coordinately regulated by metabolic checkpoints, including mTOR, AMPK, and PI3K–AKT signals, which work closely to integrate extracellular and intracellular signals (125). Although IL-33 activates the same metabolic regulators, it induces different metabolic phenotypes in different immune cell subsets. The hypothesis predicts that additional metabolic components mediated by IL-33 are necessary to induce metabolic reprogramming. Thus, it will be important to determine whether and by what mechanism IL-33 co-opts metabolic checkpoints and contributes to metabolic activity in immune cells.

With regard to IL-33-mediated epigenetic modification, the IL-33-related chromatin landscape and changes in epigenetic regulatory enzymes have only been determined in certain immune cells. However, the causal relationship between epigenetic modifications and IL-33 has not been established. Given that various metabolites are known to modulate epigenetic modifiers, a better understanding is now needed regarding the IL-33-mediated interdependence between metabolic reprogramming and epigenetic modification. In other words, it is very challenging to unambiguously demonstrate the epigenetic status of IL-33-stimulated immune cells and how this can be manipulated by IL-33 according to metabolic and functional demands.

## Author contributions

JLu conceived this study, and wrote the article. JLin, RM, and JLiu researched data, made substantial contributions to content and edited the manuscript. All authors critically read and commented on the final manuscript. All authors contributed to the article and approved the submitted version.

## Funding

This work was supported by the National Natural Science Foundation of China (82073860, 81603122) to JLu, and funding from the Young Elite Scientist Sponsorship Program of Henan Association for Science and Technology (2021HYTP048 to JLu).

## Acknowledgments

We apologize to those authors whose work could not be cited due to space limitations.

## Conflict of interest

The authors declare that the research was conducted in the absence of any commercial or financial relationships that could be construed as a potential conflict of interest.

## Publisher’s note

All claims expressed in this article are solely those of the authors and do not necessarily represent those of their affiliated organizations, or those of the publisher, the editors and the reviewers. Any product that may be evaluated in this article, or claim that may be made by its manufacturer, is not guaranteed or endorsed by the publisher.
